# Single-Stage Surgical Correction of Anorectal Malformation Associated with Rectourinary Fistula in Male Neonates

**Published:** 2013-01-01

**Authors:** Ernesto Leva, Francesco Macchini, Rossella Arnoldi, Antonio Di Cesare, Valerio Gentilino, Monica Fumagalli, Fabio Mosca, Akbar Bhuiyan, Maurizio Torricelli, Tahmina Banu

**Affiliations:** 1 Dept. of Pediatric Surgery, FONDAZIONE IRCCS CA’ GRANDA – Ospedale Maggiore Policlinico, Milan – Italy; 2 Neonatal ICU, FONDAZIONE IRCCS CA’ GRANDA – Ospedale Maggiore Policlinico, Milan – Italy; 3 Dept. of Pediatric Surgery, CHITTAGONG MEDICAL COLLEGE HOSPITAL, Chittagong – Bangladesh

**Keywords:** Anorectal malformation, Primary repair, Recto-urinary fistula

## Abstract

Introduction: The treatment of children affected by anorectal malformations (ARM) is characterized by some unsolved problems. The three-stage surgical correction has been known to be most effective in preventing complications, but recently new approaches have been proposed. We describe our experience with the newer approaches.

Methods: Twenty three male newborns, affected by ARM and recto-urinary fistula, were treated in 2 different centers in 8 years. Nineteen neonates (birth weight 2.4 - 3.5 kg) received a primary posterior sagittal anorectoplasty (PSARP) at the Department of Pediatric Surgery of the Chittagong Medical College Hospital (group 1). Four term neonates (birth weight 2.9 - 3.4 kg) received a primary pull-through with combined abdomino-perineal approach at the Pediatric Surgery Department of Fondazione Cà Granda of Milan (group 2).

Results: Among patients of Group 1, 11 patients had a recto-bulbar fistula and 8 a recto-prostatic fistula. Among the Group 2, 2 had a recto-bulbar fistula and 2 a recto-prostatic fistula. The site of fistula was decided at the time of surgery. In Group 1, 5 post-surgical complications were recorded (26%); 1 child died of sepsis, 3 had dehiscence and 1 stenosis, which resolved with dilatation. In Group 2, the only post-operative complication of small rectal prolapse resolved spontaneously after a few months on follow-up. Group 2 patients were followed-up in a dedicated multidisciplinary colorectal center.

Conclusions: Primary repair of ARMs with recto-urinary fistula is a feasible, safe and effective technique in the neonatal period. A combined abdominal and perineal approach seems to guarantee better results. A dedicated team is mandatory, both for the surgical correction and for a long-term follow-up.

## INTRODUCTION

The management of children affected by anorectal malformations (ARM) is still affected by some unsolved problems. Pena advocates 3 staged management- colostomy at birth, posterior sagittal anorectoplasty (PSARP) and closure of colostomy for males neonates affected by ARM associated with recto-urinary fistula [1-3]. This surgical approach is considered the most effective in preventing incontinence, thanks to its meticulous respect of the perineal structures [4]. In the last 2 decades, with the advent of laparoscopy, new approaches to ARMs were proposed, at first by Willital and then by Georgeson [5]. The laparoscopy was very useful in identifying the rectal wall, isolating and closing the fistula, thus facilitating the anorectoplasty. The results of two different techniques of primary repair performed in two Departments of Pediatric Surgery are here analysed: in the first group a posterior sagittal approach was chosen, while in the second one a combined abdominal and perineal correction was preferred.

## MATERIALS AND METHODS

From January 2002 to December 2009, 23 male newborns affected with ARM associated with recto-urinary fistula were treated in 2 different centers within first 48 hours of lives. 

The diagnosis of ARM with recto-urinary fistula was easily established within first 24 hours of birth. An invertogram was obtained 24 hours after birth in all the patients to determine the distance between the rectal pouch and the proposed anal site. Neonates without evidence of meconium in urine after 24 hours from birth and neonates with a delayed presentation after 48 hours from birth were excluded by the study. No other exclusion criteria, such as associated cardiac diseases, were considered.

Group 1 consisted of 19 neonates (gestational age 35 - 41 weeks; birth weight 2.4 - 3.5 kg) who underwent a primary PSARP at the Department of Pediatric Surgery of the Chittagong Medical College Hospital. Among them, 37% were born by caesarean section. Four patients had cardiac malformations (atrioseptal defect (n=2); ventriculoseptal defect (n=1); pulmonary stenosis (n=1)), while 9 had genito-urinary malformations (vesicoureteral reflux (n=4); hypospadias (n=3); multicystic kidney (n=1) and 1 ectopic kidney (n=1).

Group 2 consisted of 4 term neonates (birth weight 2.9 - 3.4 kg) who received a primary pull-through with combined abdomino-perineal approach at the Pediatric Surgery Department of Fondazione Cà Granda of Milan, Italy. All of them had a spontaneous delivery and a regular post-natal course. There were no associated cardiac and renal malformations in this sub-group. The study of lumbo-sacral anomalies and intra-spinal defects was postponed; X-ray of sacrum and spinal US or MRI was done before age of 2 months. For Group 2, continence was evaluated on the basis of the Krickenbeck consensus statement [6].

In Group 1, PSARP and submucosal dissection were performed according to Pena’s technique. With a posterior sagittal approach, the muscle complex was open, the rectum was reached and the urinary fistula was ligated, and then an anorectoplasty was performed. 

In Group 2, through a pfannenstiel incision, the abdomen was explored. The dilated descending colon was opened, decompressed from the meconium and subsequently closed. Rectal dissection begins at the peritoneal reflection and this was facilitated by an antero-superior traction applied to the empty bowel remaining close to the bowel wall. The dissection and ligation of the fistula resulted easy in the present population of newborns. Legs were elevated. Pena’s muscle stimulator was used to determine the correct anal site. Vertical incision with a complete visualisation of the muscle complex was performed, a Step Verres needle was passed through the muscle complex and dilatations of the new anal canal to Hegar number 11 were done. After this, the rectum was pulled through and the anoplasty was performed.

## RESULTS

In Group 1 (n=19), 11 patients had a recto-bulbar fistula and 8 a recto-prostatic fistula. In Group 2 (n=4), 2 had a recto-bulbar fistula and 2 a recto-prostatic fistula.

The site of fistula was decided at the time of surgery. A good dissection of the rectal pouch, isolation and ligation of the fistula and a correct anorectoplasty was achieved in all patients.

Average duration of the operation was 2 hours and 10 minutes (range: 1h50min - 3h30min) in Group 1 and 2 hours and 35 minutes (range: 2h 20min - 3h 10min) in Group 2. 

No intra-operative and anaesthesia complications were recorded in either centers. Feeding was started on day 5 in Group 1 and on day 12 in Group 2. All neonates passed meconium after an average of 48 hours post-surgery.

In Group 1, 5 post-surgical complications were recorded (26%); 1 child died after 3 days from surgery for sepsis, 3 presented with dehiscence within 48 hours from surgery (one severe, which required colostomy and two successfully solved with conservative treatment), and 1 developed stenosis which required prolonged dilatation. Two patients were lost to follow-up. Sixteen patients were toilet trained and reached regular bowel movements and no fecal incontinence in a mid-term follow-up of 3 years. Five out of these patients had vertebral dysraphism (such as anomalies in number and form of vertebrae), but none had neurospinal defects (such as tethered cord, or intradural lipoma). None of these boys had any urinary complaints; no voiding cystourethrograms (VCUG) were done.

In Group 2, only 1 surgical complication was recorded, consisting in a small rectal prolapse, which spontaneously resolved on follow-up after a few months. None of the patients presented strictures or dehiscence in the short term follow-up and the quality of life was considered good for all the children as related by parents. All these patients achieved regular bowel movements. All these patients achieved regular bowel movements and no soiling in a mid-term follow-up of 3 years was recorded, while 2 of them needed daily laxative for a stasis of grade 2 according to Krickenbeck consensus statement. Patients of this group were followed-up in a Multidisciplinary Colorectal Center (mean time of follow-up of 3 years), with regular ambulatory evaluations by a dedicated team of specialist, formed by neurosurgeons, urologists, gynaecologists, psychologists and pediatric surgeons. None of them presented associated spinal defects. VCUG were normal in all boys.

## DISCUSSION

ARM comprise a spectrum of diseases ranging from minor defects, characterized by a good functional prognosis, to more complex ones, often associated with other anomalies with poor functional prognosis [1-3]. The management of these children is still affected by some unsolved problems.

The most important advancement in the treatment of ARMs came from Pena in the early 1980s, with the introduction of a new operative approach (PSARP), effective for the entire spectrum of ARMs [7]. The well-known technique consists in dissecting the external anal sphincter complex and the levator muscle, by staying carefully in the midline and thus avoiding lesions of the surrounding structures. In particular, this technique needs a 3 stage-approach for the treatment of recto-urinary fistula in male, consisting in a colostomy at birth, the PSARP repair within 2 months and a subsequent closure of colostomy [1, 3]. Over the years, the crucial role of the muscle complex in achieving continence and preserving the sensory and as motor functions became evident. In the early 1990s, a minimal invasive technique for correction of ARM was proposed for children with recto-urinary fistula by Willital, later popularized by Georgeson [5]. 

In past years, a few papers describing single-stage procedure at birth have been published [8-10]; these were received with some criticism by the international experts of the disease. The proposal consisted in performing a single surgical procedure, unifying the “anatomical criteria” of Pena and the innovations brought by minimal invasive surgery [5].

Here, the experiences of 2 different centers on the primary repair of ARMs with recto-urinary fistula are reported. At the Department of Pediatric Surgery of the Chittagong Medical College Hospital, the decision to treat males affected by ARM with recto-urinary fistula by a primary repair was taken in consideration of the limited facilities of the area and of the difficulties of a staged surgical program in children living in suburban villages. Post-surgical complications were especially related to the lack of availability of antibiotics, parenteral nutrition and laboratory tests. On the contrary, in a mid-term follow-up, all children presented satisfactory results. 

Following the experience of the team from Bangladesh, a single-stage surgical approach was applied to 4 male children affected by ARM with recto-urinary fistula in the Department of Pediatric Surgery of Policlinico of Milan (Group 2). Post-surgical complications were few and self-limited. 

Based on the present experience, few facts have to be taken in consideration.

Appropriate selection criteria have to be decided for the newborns that would be subjected to primary repair. The main objective evidence is the presence of meconium in the urine, as sign of recto-urinary fistula. The fistula limits the risk to search the rectum somewhere, allowing surgeons to follow the anatomical structures, to isolate the rectum and the fistula and to reconstruct the anatomy of the low pelvic floor and perineum with the same principles and dogmas of posterior sagittal approach. If there is no evidence of meconium in the urine or in the perineum after 24 hours from birth in male newborns, staged management is advocated. The invertogram was realised to be a futile investigation as the surgical approach did not change on the fact that how high the rectal pouch ended.

The satisfactory results, in terms of post-surgical complications, were obtained taking extreme care to respect some fundamental principles: to dissect the perineum close to the rectum in order to avoid injuries to the pelvic autonomic nerve of bladder and penis; to isolate carefully the recto-urinary fistula, taking attention not to damage the urethra; to dilate progressively the perineum to avoid strictures; to perform the anoplasty with the same principles of PSARP, included the use of the Pena stimulator to determine the anal site [7].

According to these surgical criteria, a combined abdominal and perineal approach seems to guarantee a better isolation of the rectum and the fistula and, as a consequence, a lower rate of surgical complications. 

It is clear that a primary repair represent an attractive choice for the treatment of ARMs. In fact, it reduces the number of operations from 3 to 1, with the obvious advantages in terms of surgical and anaesthesia risks; it avoids colostomy, with lower surgical complications and familiar discomfort in terms of management, psychological and economic burdens [8]; it allows the passage of stools since the beginning, with a theoretical early establishment of the brain-defecation reflexes [9, 10]. Furthermore, a precocious closure of the fistula can avoid continued urinary tract colonization through it [10]. 

Review of the literature did not reveal any experience of laparoscopy in the single-stage repair of ARMs during the neonatal period. We found that laparotomy was effective in isolating distally the fistula in these newborns. Furthermore, it may represent a valid approach in case of dilated rectal pouch, allowing an enterotomy and the emptying of the distal bowel (never needed in the present population). 

If further studies will demonstrate that the results of the primary repair are similar to that of the traditional approach in terms of functional outcome, the primary repair will represent a valid option for surgeons trained and dedicated to the surgery of ARM.

## CONCLUSION

Primary repair of ARMs with recto-urinary fistula is a feasible, safe and effective technique in the neonatal period. A combined abdominal and perineal approach seems to guarantee better results. Further studies are needed to evaluate if the incidence of surgical and functional complications is similar to the traditional staged management. The role of a dedicated team is mandatory, both for the surgical correction and for a long-term follow-up.

## Footnotes

**Source of Support:** Nil

**Conflict of Interest:** None
